# Microbial Small Talk: Volatiles in Fungal–Bacterial Interactions

**DOI:** 10.3389/fmicb.2015.01495

**Published:** 2016-01-05

**Authors:** Ruth Schmidt, Desalegn W. Etalo, Victor de Jager, Saskia Gerards, Hans Zweers, Wietse de Boer, Paolina Garbeva

**Affiliations:** ^1^Department of Microbial Ecology, Netherlands Institute of EcologyWageningen, Netherlands; ^2^Department of Soil Quality, Wageningen UniversityWageningen, Netherlands

**Keywords:** soil microorganisms, fungal–bacterial interactions, volatiles, terpenes, signaling, motility

## Abstract

There is increasing evidence that volatile organic compounds (VOCs) play an important role in the interactions between fungi and bacteria, two major groups of soil inhabiting microorganisms. Yet, most of the research has been focused on effects of bacterial volatiles on suppression of plant pathogenic fungi whereas little is known about the responses of bacteria to fungal volatiles. In the current study we performed a metabolomics analysis of volatiles emitted by several fungal and oomycetal soil strains under different nutrient conditions and growth stages. The metabolomics analysis of the tested fungal and oomycetal strains revealed different volatile profiles dependent on the age of the strains and nutrient conditions. Furthermore, we screened the phenotypic responses of soil bacterial strains to volatiles emitted by fungi. Two bacteria, *Collimonas pratensis* Ter291 and *Serratia plymuthica* PRI-2C, showed significant changes in their motility, in particular to volatiles emitted by *Fusarium culmorum.* This fungus produced a unique volatile blend, including several terpenes. Four of these terpenes were selected for further tests to investigate if they influence bacterial motility. Indeed, these terpenes induced or reduced swimming and swarming motility of *S. plymuthica* PRI-2C and swarming motility of *C. pratensis* Ter291, partly in a concentration-dependent manner. Overall the results of this work revealed that bacteria are able to sense and respond to fungal volatiles giving further evidence to the suggested importance of volatiles as signaling molecules in fungal–bacterial interactions.

## Introduction

In terrestrial ecosystems fungi and bacteria live in complex multi-species networks (Frey-Klett et al., 2011; Hung et al., 2015). Within those networks, both fungi and bacteria produce a plethora of secondary metabolites of diverse chemical classes (Schulz and Dickschat, 2007; Morath et al., 2012; Muller et al., 2013). Several of these secondary metabolites are diffusible molecules, such as antibiotics and antibiotic-like substances or signaling molecules, which are important in interactions between fungi and bacteria (Ryan and Dow, 2008; Frey-Klett et al., 2011; Haq et al., 2014).

A group of metabolites that is increasingly recognized to play important roles in microbial interactions and communications are volatile organic compounds (VOCs). Those compounds are low molecular weight carbon-containing compounds (<400 Da) that evaporate easily at normal temperatures and air pressures (Schulz and Dickschat, 2007; Bitas et al., 2013). Their physico-chemical properties facilitate evaporation and diffusion through both water- and gas-filled pores in soil and rhizosphere environments (Schmidt et al., 2015). Hence, they possess important functions for long distance fungal–bacterial interactions in the porous soil matrix.

Although it is known that many soil microorganisms produce a wide range of volatile compounds (Wheatley, 2002; Effmert et al., 2012) relatively little attention has been paid to fungal volatiles and to their ecological role. In these studies, over 300 distinct volatiles have been identified from fungi, belonging to different chemical classes including alcohols, benzenoids, aldehydes, alkenes, acids, esters, and ketones (Morath et al., 2012; Piechulla and Degenhardt, 2014). However, most research focused on volatiles produced by single species growing under nutrient rich conditions which is far from the nutrient-limited growth that most microbes experience in soil (Garbeva and de Boer, 2009; Kai et al., 2009; Weise et al., 2012). Furthermore, it has been shown that the composition of volatiles can vary depending on several factors, such as the fungal growth stage, moisture, temperature and pH (Wheatley, 2002; Insam and Seewald, 2010; Romoli et al., 2014).

Within the past years, it has become evident that microbial volatiles can play two major roles in long-distance interactions within soil microbial communities: (i) as infochemical molecules affecting the behavior, population dynamics and gene expression in responding microorganisms and (ii) as interference competition tools suppressing or eliminating potential enemies (Effmert et al., 2012; Garbeva et al., 2014a,b; Schmidt et al., 2015). Currently, most research on microbial volatiles is focused on the effect of bacterial volatiles on other bacteria and/or fungi whereas the effect of fungal volatiles on bacteria remains largely unknown.

In this study, we aimed to profile volatiles emitted by a range of fungal and oomycetal soil strains and to test the effect of these volatiles on the behavior of phylogenetically different soil bacteria which are known from previous studies to interact with fungi (Leveau et al., 2010; Mela et al., 2011; Garbeva et al., 2014b). The main research questions we addressed were (1) Can bacteria sense the presence of fungal and oomycetal volatiles and react with specific phenotypical responses and (2) Is the response dependent on the nutrient conditions and growth stage of the fungal and oomycetal strains?

### Materials and Methods

### Bacterial, Fungal, and Oomycetal Strains and Growth Conditions

All bacterial, fungal and oomycetal strains used in this study (**Table 1**) have been isolated from bulk or rhizosphere soil. The bacterial strains *Collimonas fungivorans* Ter331 and Ter6, *C. pratensis* Ter91, Ter291, and *C. arenae* Ter10 and Ter282, *Burkholderia* sp. AD24, *Pedobacter* sp. V48 and *Paenibacillus* sp. AD87 are strains from sandy dune soils in The Netherlands (De Boer et al., 1998; de Boer, 2004). *Serratia plymuthica* PRI-2C strain was isolated from maize rhizosphere, The Netherlands (Garbeva et al., 2004). Bacterial strains were pre-cultured from frozen glycerol stocks on 0.1 Tryptic Soya Broth plates (0.1 TSB; 5 g L^-1^ NaCl, 1 g L^-1^ KH_2_PO_4_, 3 g L^-1^ TSB, 20 g L^-1^ Merck Agar; pH = 6.5; Garbeva and de Boer, 2009) and grown for 3 days at 20°C prior usage.

**Table 1:** Bacterial, fungal, and oomycetal strains used in this study.

	Phylum/Class	Source	Accession number	Reference
**Bacterial strains**				
*Collimonas fungivorans* Ter331	Proteobacteria, β-Proteobacteria	Inner coastal dune soil in Terschelling, the Netherlands	NR_074756	De Boer et al., 1998; de Boer, 2004
*Collimonas fungivorans* Ter6	Proteobacteria, β-Proteobacteria	Inner coastal dune soil in Terschelling, the Netherlands	CP013232	De Boer et al., 1998; de Boer, 2004
*Collimonas pratensis* Ter91	Proteobacteria, β-Proteobacteria	Inner coastal dune soil in Terschelling, the Netherlands	CP013234	De Boer et al., 1998; de Boer, 2004
*Collimonas pratensis* Ter291	Proteobacteria, β-Proteobacteria	Inner coastal dune soil in Terschelling, the Netherlands	CP013236	De Boer et al., 1998; de Boer, 2004
*Collimonas arenae* Ter10	Proteobacteria, β-Proteobacteria	Inner coastal dune soil in Terschelling, the Netherlands	CP013233	De Boer et al., 1998; de Boer, 2004
*Collimonas arenae* Ter282	Proteobacteria, β-Proteobacteria	Inner coastal dune soil in Terschelling, the Netherlands	CP013235	De Boer et al., 1998; de Boer, 2004
*Burkholderia* sp. AD24	Proteobacteria, β-Proteobacteria	Rhizosphere and bulk soil of *C. arenaria*	KJ685239	De Ridder-Duine et al., 2005
*Paenibacillus* sp. AD87	Firmicutes, Bacilli	Rhizosphere and bulk soil of *C. arenaria*	KJ685299	De Ridder-Duine et al., 2005
*Pedobacter* sp. V48	Bacteroidetes, Sphingobacteriia	Coastal dune soil, the Netherlands	DQ778037	de Boer et al., 2007
*Serratia plymuthica* PRI-2C	Proteobacteria, y-Proteobacteria	Maize rhizosphere soil, the Netherlands	AJTB00000000	Garbeva et al., 2004
**Fungal and Oomycetal strains**				
*Trichoderma harzianum* PVDG2	Ascomycota	Coastal dune soil, the Netherlands	KC888990	De Boer et al., 1998
*Fusarium culmorum* PV	Ascomycota	Coastal dune soil, the Netherlands	KT992460	De Boer et al., 1998
*Verticillium dahliae* JR2	Ascomycota	Tomato soil, Canada	PRJNA175765	Huang, 2014
*Mucor hiemalis* PVDG1	Zygomycota	Coastal dune soil, the Netherlands	KC888987	De Boer et al., 1998
*Rhizoctonia solani* AG2.2IIIB	Basidiomycota	Sugar beet rhizosphere soil, the Netherlands	KT124637	Garbeva et al., 2014b
*Pythium ultimum* P17	Oomycota	Rhizosphere of bulb, the Netherlands	KT124638	Garbeva et al., 2014b

The fungal strains *Trichoderma harzianum* PVDG2, *Mucor hiemalis* PVDG1 and *Fusarium culmorum* PV were also isolated from a sandy dune soil in The Netherlands (De Boer et al., 1998). *Verticillium dahliae* JR2 was isolated from tomato, Canada and *Rhizoctonia solani* AG2.2IIIB was isolated from sugar beet, the Netherlands (Garbeva et al., 2014b). The fungal oomycete *Pythium ultimum* P17 was isolated from tulip bulb rhizosphere, The Netherlands (Garbeva et al., 2014b). All fungal and oomycetal strains were pre-cultured on 0.5 Potato Dextrose Agar plates (19.5 g L^-1^ PDA, 7.5 g L^-1^ Merck Agar; pH = 5.5–6; Fiddaman and Rossall, 1993) and incubated for 6 days at 20°C prior usage.

### Screening for Volatile-Mediated Phenotypes

To investigate the effect of fungal volatiles on bacterial phenotypes, variations of assays in double plate-within-a-plate system (Lee et al., 2015) were performed (schematically described in **Supplementary Figure S1**). A 3.5-cm Petri dish containing the fungal and oomycetal strains was placed into a partitioned 9-cm Petri dish containing the bacterial strains. Plates containing only sterile medium was used as a control. The bacterial response to fungal and oomycetal volatiles was studied by comparing the phenotypic responses of the bacteria under the two nutrient conditions.

### Test of Bacterial Growth and Antimicrobial Activity

The 3.5-cm Petri dish contained either 3 mL 0.5 PDA medium or 1.5% water-agar (5 g L^-1^ NaCl, 1 g L^-1^ KH_2_PO_4_, 15 g L^-1^ Merck Agar; pH 6.5) supplied with artificial root exudates (WA + ARE). The artificial root exudates stock solution contained 18.4 mM glucose; 18.4 mM fructose; 9.2 mM saccharose; 4.6 mM citric acid; 9.2 mM lactic acid; 6.9 mM succinic acid; 18.4 mM L-serine; 11 mM L-glutamic acid and 18.4 mM L-alanine (C/N 10.4). Per liter of water-agar, 70.4 mL of ARE stock solution was added. A small plate (3.5 cm) containing the fungal and oomycetal plugs (6 mm in diameter) was placed into the partitioned Petri dish (9 cm) and grown for 3 days at 20°C (**Supplementary Figure S1A**). Bacterial strains were grown in 10 mL 0.1 TSB broth overnight at 20°C. The cells were washed twice with sterile 10 mm sodium phosphate buffer (1.361 g KH_2_PO_4_ in 1 L milliQ, pH 6.5), adjusted to a range of 1 × 10^6^–10^2^ cells/mL, 5 μl of cell suspension was spotted on 1.5% WA + ARE of the partitioned Petri dish containing the fungal and oomycetal strains in the other compartment. The Petri dish was then closed and incubated for 3 days at 20°C. On day 6, bacterial growth was determined by comparing the cfu/mL of bacteria exposed to fungal and oomycetal volatiles to that of bacteria exposed only to sterile growth media only.

To test the triggering of antimicrobial activity by bacterial strains in response to fungal and oomycetal volatiles, an agar overlay assay was performed on day 6 (Tyc et al., 2014). The two indicator organisms *Escherichia coli WA321* and *Staphylococcus aureus 533R4* were grown in liquid LB broth overnight at 37°C, 220 rpm. Fresh LB-agar (1.5% Merck Agar) was prepared, cooled down to ∼45°C and the target organisms were added to 6 × 10^5^ cells/mL (*E. coli* WA321) or 4 × 10^5^ cells/mL (*S. aureus* 533R4). A volume of 5 mL liquid LB-agar containing the target organisms was poured over the compartment in which bacteria were growing. After solidification of the overlay agar, plates were incubated overnight at 37°C. The next day, plates with bacteria exposed to fungal and oomycetal volatiles were examined for visible zones of inhibition (ZOI) compared to the bacteria exposed only to sterile media.

### Test of Bacterial Motility

The effect of fungal and oomycetal volatiles on bacterial swarming and swimming motility was assessed on soft WA + ARE [0.6% wt/vol and 0.3% wt/vol, adapted from de Bruijn and Raaijmakers (2009)]. After autoclaving, the medium was cooled down in a water bath to 60°C. Next, 10 mL of the medium was pipetted into the partitioned Petri dish and the plates were kept for 24 h at room temperature (20°C) prior to the swarming and swimming assay. For all swarming and swimming assays, the same conditions (agar temperature and volume, time period of storage of the poured plates) were kept constant to maximize reproducibility. A plate containing the fungal and oomycetal plugs (6 mm in diameter) that were grown on either 0.5 PDA or 1.5% water-agar supplied with artificial root exudates (WA + ARE) was placed into the partitioned Petri dish and grown for 3 days at 20°C (**Supplementary Figure S1B**). Recipient bacteria were grown in 10 mL 0.1 TSB broth overnight at 20°C. The cells were washed twice with sterile 10 mm sodium phosphate buffer (1.361 g KH_2_PO_4_ in 1 L milliQ, pH 6.5), adjusted to 1 × 10^7^ cells/mL and 5 μl of cell suspension was spotted in the center of the soft WA + ARE of the partitioned Petri dish containing the fungal and oomycetal strains. The Petri dish was then closed and incubated for 3 days at 20°C. On day 6, volatile effect was determined by comparing the motility diameter of bacteria exposed to fungal and oomycetal volatiles to that exposed only to media. Motility diameter was calculated by measuring the radial swimming and swarming zones of the bacteria in two directions and calculating the mean for each of the three replicates.

### Test of Bacterial Biofilm Formation

The test for biofilm formation was adapted and modified from O’Toole et al. (1999). A small plate containing the fungal and oomycetal plugs (6 mm in diameter) that were grown on either 0.5 PDA or 1.5% water-agar supplied with artificial root exudates (WA + ARE) was placed into the partitioned Petri dish and grown for 3 days at 20°C (**Supplementary Figure S1C**). Recipient bacteria were grown in 10 mL 0.1 TSB broth overnight at 20°C. The cells were washed twice with sterile 10 mm sodium phosphate buffer, adjusted to 1 × 10^7^ cells/mL and 20 μl of cell suspension was added into six-wells strip of a flat-bottom 96-well plates made of transparent polystyrene (Greiner) with 180 μl 0.1 TSB broth per well. Part of the 96-well plates was placed into the partitioned Petri dish containing the fungal and oomycetal strains in the other compartment. The Petri dish was then closed and incubated for 2 days at 20°C. On day 6, the six-well strip was removed from the large Petri-dish and 10 μl of 1% crystal violet solution was added to each well. These were incubated for 15 min at room temperature and rinsed three times with demi water. Biofilm formation was estimated by solubilization of crystal violet by adding 200 μl of 96% ethanol and determining the OD600. Volatile activity was determined by comparing biofilm formation from bacteria exposed to fungal and oomycetal volatiles to that of bacteria exposed to media.

### Fungal and Oomycetal Volatile Trapping and GC-Q-TOF Analysis

For the collection of fungal and oomycetal volatiles, glass Petri dishes with leads to which a steel trap containing 150 mg Tenax TA and 150 mg Carbopack B (Markes International Ltd., Llantrisant, UK) could be fixed were used (Garbeva et al., 2014a). Fungi/oomyctes were grown on either 0.5 PDA or 1.5% water-agar supplied with artificial root exudates (WA + ARE) for 3 and 6 days at 20°C. Petri dishes containing medium only served as controls. All treatments were inoculated in triplicates. The Tenax steel traps were collected at two time points for two fungal and oomycetal growth stages (days 3 and 6) and under two nutrient conditions. Traps were removed, capped and stored at 4°C until analysis using GC-Q-TOF. Volatiles were desorbed from the traps using an automated thermal desorption unit (model UnityTD-100, Markes International Ltd., Llantrisant, UK) at 210°C for 12 min (He flow 50 mL/min) and trapped on cold trap at -10°C. The trapped volatiles were introduced into the GC-QTOF (model Agilent 7890B GC and the Agilent 7200A QTOF, Santa Clara, CA, USA) by heating the cold trap for 3 min to 280°C with split ratio set to 1:20. The column used was a 30 mm × 0.25 mm ID RXI-5MS, film thickness 0.25 μm (Restek 13424-6850, Bellefonte, PA, USA). Temperature program used was as follows: 39°C for 2 min, from °C to 95°C at 3.5°C/min, then to 165°C at 6°C/min, to 250°C at 15°C/min and finally to 300°C at 40°C/min, hold 20 min. The Volatiles were detected by the MS operating at 70 eV in EI mode. Mass spectra were acquired in full scan mode (30–400 amu, 4 scans/s). MassHunter Qualitative Analysis Software V B.06.00 Build 6.0.633.0 (Agilent Technologies, Santa Clara, CA, USA) was used to control the instrument and for data acquisition and analysis. The mass chromatogram that were generated exported as mzData files and were processed (peak picking, baseline correction and peak alignment) in untargeted manner using the MetAlign software package (Lommen, 2009). Extraction and reconstitution of compound mass spectra were performed according to the method described previously by Tikunov et al. (2012). Identification of metabolites was performed using NIST-MS Search and accurate mass, retention indices, and spectra match factor using NIST 2014 V2.20 (National Institute of Standards and Technology, USA, http://www.nist.gov) and Wiley ninth edition spectral libraries and by their linear retention indexes (lri). The lri values were compared with those found in the NIST and in the in-house NIOO lri database.

### Test of Terpene Compounds on Bacterial Motility

Four fungal volatiles, α-Terpinene, β-Phellandrene, 3-Carene, and Camphene were confirmed though injection of authentic standards obtained from Sigma–Aldrich and the Natural Products Laboratory, Leiden. Volatile chemicals were dissolved in ethanol with concentrations of 10 nM, 100 nM, 10 μM, 100 μM, 10 mM, and 100 mM. The effect of individual terpene volatiles on the motility of *C. pratensis* Ter291 and *S. plymuthica* PRI-2C was investigated using the double plate-within-a-plate system as described previously. The pure compounds were applied as a 10-μl droplet on a sterile filter paper (1 cm × 1 cm) on the bottom of a 3.5 cm Petri dish which was then transferred into the partitioned Petri dish. Plates were sealed immediately and incubated for 3 days at 20°C. The activity of the pure compounds was determined by comparing the motility diameter of bacteria exposed to pure volatile compounds as single compounds to that of bacteria exposed to control (only ethanol). Motility diameter was calculated by measuring the radial swimming and swarming zones of the bacteria in two directions and calculating the mean for each of the three replicates.

### Statistical Analysis

In all experiments, both for the treatments and the controls three independent replicates were considered. For the metabolomics analyses Genemaths XT software (Applied Maths, Belgium) was used for ANOVA (with Bonferroni correction), Principal Component Analysis (PCA) and Hierarchical Cluster Analysis. Pearson’s correlation coefficients were used to calculate the distance or similarity between two entries and the resulting clusters were summarized using a complete linkage algorithm. The raw values of each sample were log-transformed and auto-scaled by the use of the average as an offset and the standard deviation as scale [raw value-average (offset)/SD (scale)].

Data obtained from the phenotypical assays were expressed as standard error of the mean and analyzed using OriginPro 2015 (OriginLab Corporation, MA) and SPSS (Science Inc., IL). Student’s *t*-test (*p* < 0.05) and one-way analysis of variance (ANOVA) between groups (treatments and control) were performed for all data.

## Results

### Volatile Profiles of Fungal and Oomycetal Strains

**FIGURE 1:**
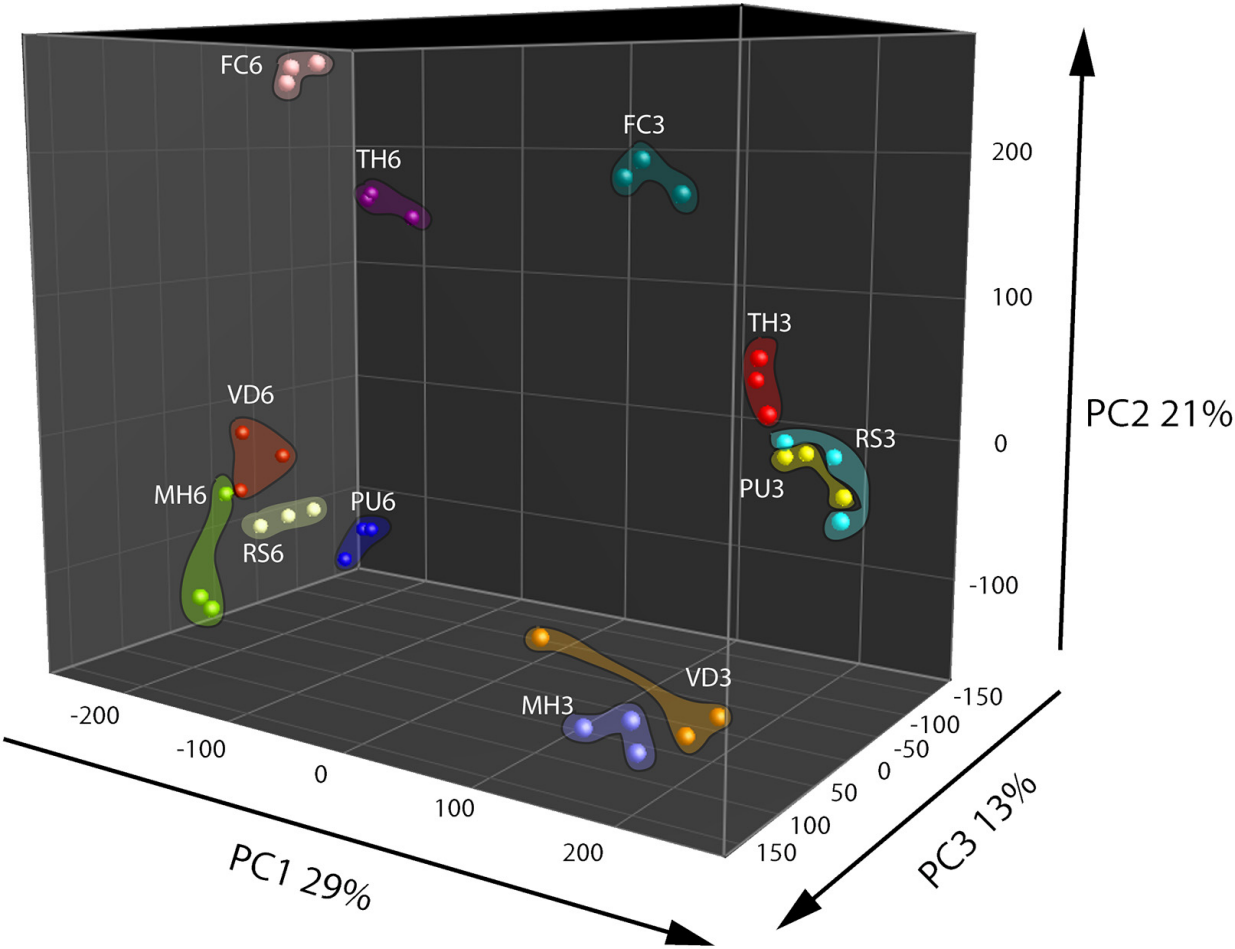
**Principal Component Analysis (PCA) of fungal and oomycetal strains based on 106 volatiles that were significantly different (*P* < 0.05 with Bonferroni correction) in abundance between at least two strains when grown on water agar supplied with artificial root exudates at day 3 (early growth stage) and day 6 (late growth stage).** MH, *Mucor hiemalis*; RS, *Rhizoctonia solani*; PU, *Pythium ultimum*; VD, *Verticillium dahliae*; FC, *Fusarium culmorum*; TH, *Trichoderma harzianum*.

Based on metabolomics analysis a total of 306 putative volatiles were detected when the fungal and oomycetal strains were grown on the nutrient poor WA + ARE medium. 106 of these volatiles differed significantly in abundance between at least two of the fungal/oomycetal strains. A total of 578 putative volatiles were detected from the head space of the fungal and oomycetal strains when grown on the nutrient rich PDA with 173 volatiles significantly different in their abundance between at least two fungal/oomycetal strains. Volatiles that differed significantly were further used to compute PCA and Hierarchical Cluster Analysis (HCA). In the PCA, the first three principal components (PC) explained 63% of the total variation observed between the fungal/oomycetal strains that were grown on nutrient poor medium (**Figure 1**). The first principal component PC1 explained 29% of the total variation and is primarily attributed to volatiles that were altered depending on the growth stage of the fungal/oomycetal strains (**Figure 1**). The emission profile of these volatiles is indicated in cluster 5 of the HCA and is characterized by volatiles with higher abundance at the early stage of growth in all strains (**Supplementary Figure S2A**). Volatiles in cluster 4 of the HCA also showed higher emission in four out of the six strains (namely *M. hiemalis*, *R. solani*, *T. harzianum*, *F. culmorum*) at the early stage of growth while their emission pattern and abundance was fairly similar for the remaining two strains (*P. ultimum* and *V. dahliae*) at both growth stages. The second PC explained 21% of the total variation and is attributed to the volatiles indicated in clusters 6 and 7 of the HCA (**Figure 1** and **Supplementary Figure S2A**, clusters 6 and 7). Volatiles in cluster 6 are primarily consisting of terpenes and were largely produced by *F. culmorum* at both growth stages with increased emission at the later growth stage (**Supplementary Figure S2A**). Volatiles in cluster 7 were abundantly detected in *F. culmorum* and *T. harzianum* at both growth stages with a noticeable increase in emission at the later growth stage. Some volatiles within this cluster were also detected in *V. dahliae* and were increasingly abundant at the later growth stage (**Supplementary Figure S2A**, cluster 7). The third PC explained 13% of the total variation. The volatiles that explained this variation are indicated in cluster 2 of the HCA and are mainly emitted by *P. ultimum* at the later stage of growth (**Supplementary Figure S2A**, cluster 2).

**FIGURE 2:**
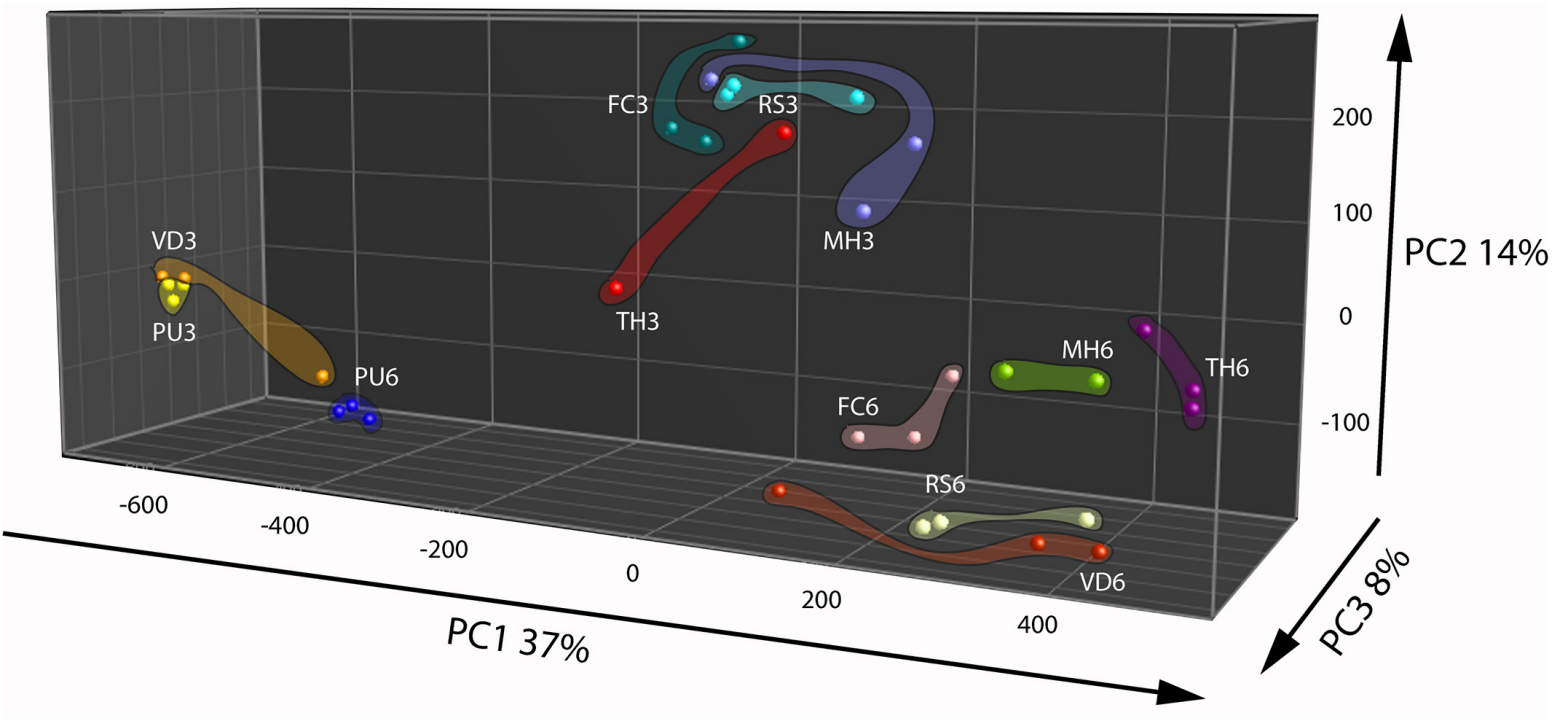
**Principal Component Analysis of fungal and oomycetal strains based on 173 volatiles that were significantly different (*P* < 0.05 with Bonferroni correction) in their abundance at least between two strains when grown on potato dextrose agar at day 3 (early growth stage) and day 6 (late growth stage).** MH, *Mucor hiemalis*; RS, *Rhizoctonia solani*; PU, *Pythium ultimum*; VD, *Verticillium dahliae*; FC, *Fusarium culmorum*; TH, *Trichoderma harzianum*.

Principal Component Analysis based on volatiles from the fungi and oomycete grown on nutrient rich PDA showed that the first three PCs explained 60% of the total observed variation between the strains (**Figure 2**). The first PC that explained 37% of the total variation is related to compounds that are found in cluster 3 of the HCA (**Figure 2** and **Supplementary Figure S2B**, cluster 3). This cluster is characterized by higher emission of the volatiles at the early growth stage by all strains except *P. ultimum* and *V. dahlia*. The second PC explained 14% of the variation and is primarily associated to volatiles grouped in the cluster 6 of the HCA that predominantly explains the growth stage dependent emission of volatiles by the strains (**Figure 2** and **Supplementary Figure S2B**, cluster 6). Similar to the observation under the nutrient poor conditions, this group of volatiles showed higher emission at the early growth stage in all the strains. The third PC explained 8% of the total variation and the volatiles associated to this variation are indicated in cluster 2 of the HCA (**Figure 2** and **Supplementary Figure S2B**, cluster 2). This cluster consisted of terpenes and they are emitted predominantly by *F. culmorum* at the later growth stage. Although these terpenes and other volatiles in this cluster were emitted by *F. culmorum* on both media, they were emitted to higher extent at the early stages of growth on WA + ARE (**Supplementary Figure S2A**, cluster 6 and **Supplementary Figure S2B**, cluster 2).

The volatiles belonging to the unique clusters of *F. culmorum* were investigated in more detail for both nutrient poor and nutrient rich conditions. Cluster 6 consisted of 19 volatiles belonging to the classes of terpenes (monoterpenes and sesquiterpenes), alkylbenzenes, cycloalkenes and furans and Cluster 2 consisted of 17 volatiles belonging to the classes of terpenes (monoterpenes and sesquiterpenes), alkaloids, benzenoids and furans (**Table 2**). Some volatiles could not be identified and are thus indicated as unknown compounds with their respective retention times and accurate masses. In both clusters terpenes represented the most abundant class with unique volatiles for both nutrient conditions. The identity of the terpenes α-Terpinene, β-Phellandrene, 3-Carene, and Camphene were confirmed with commercially available authentic standards.

**Table 2:** Characteristics of volatile compounds of cluster 6 (water agar supplied with artificial root exudates) and 2 (potato dextrose agar) emitted by *F. culmorum*.

#	Compound	MSI^∗^	Average RT^∗∗^ (min)	Accurate mass	RI^∗∗∗^	Class
**Cluster 6 WA + ARE**						
(1)	2-Furancarboxaldehyde	2	4.73		771	Furans
(2)	Unknown		12.41	77.038		
(3)	α-Phellandrene	2	13.61		1005	Monoterpenes
(4)	Pentamethylcyclopentadiene	2	13.88		1006	
(5)	3-Carene	1	14.33		1017	Monoterpenes
(6)	o-Cymene	2	14.42		1026	Alkylbenzenes
(7)	Unknown	2	21.48	93.067	1197	
(8)	Unknown		27.60	93.068		
(9)	Unknown		29.22	229.001		
(10)	α-Copaene	2	29.52		1433	Sequiterpenes
(11)	1,3-Cyclopentadiene-1-butanenitrile, α-ethyl-	2	29.73		1433	Cycloalkenes
(12)	Unknown		29.84	161.128		
(13)	(-)-Isoledene	2	30.38		1472	Sequiterpenes
(14)	Unknown		30.51	93.067		
(15)	Unknown		31.09	67.053		
(16)	Unknown		31.83	80.059		
(17)	*cis*-Farnesol	2	32.59		1503	Sequiterpenes
**Cluster 2 PDA**						
(1)	2,4-Dimethylfuran	2	4.08		714	Furans
(2)	Unknown		4.88	95.047		
(3)	Camphene	1	12.79		970	Monoterpenes
(4)	α-Terpinene	1	13.85		1004	Monoterpenes
(5)	β-Phellandrene	1	14.55		1032	Monoterpenes
(6)	1,3,8-p-Menthatriene	2	19.03		1136	Sesquiterpenes
(7)	2,6-Dichloroanisol	2	20.65		1157	Benzenoids
(8)	Unknown		25.42	189.164		
(9)	Unknown		27.76	121.097		
(10)	Longifolene	2	27.91		1347	Sesquiterpenes
(11)	Ledene	2	27.98		1348	Sesquiterpenes
(12)	Y-Muurolene	2	28.28		1356	Sesquiterpenes
(13)	Streptazone C	2	29.47		1411	Alkaloids
(14)	Germacrene-D	2	29.77		1433	Sesquiterpenes
(15)	δ-Guaiene	2	30.04		1412	Sesquiterpenes
(16)	Unknown		30.31	105.068		
(17)	Unknown		30.67	67.054		
(18)	Unknown	2	31.77	109.100	1471	
(19)	α-Bisabolene	2	32.55		1500	Sesquiterpenes

*^∗^1: Identified metabolites based on authentic standards; 2: Putatively annotated compounds (e.g., without chemicals reference standards, based upon physicochemical properties and/or spectral similarity with public/commercial NIST 2014 V2.20 and Wiley ninth edition spectral libraries). The reporting grades (1 and 2) are assigned according to the proposed minimum reporting standards for chemical analysis [metabolomics standards initiative (MSI)] (Sumner et al., 2007).*

*^∗∗^Retention time.*

*^∗∗∗^Retention index.*

### Screening for Bacterial Phenotypes in Response to Fungal and Oomycetal Volatiles

A screening using variations of assays of a double-plate-within-a-plate system was performed to test phenotypical responses of 10 bacterial strains to volatiles emitted by six fungal and oomycetal strains. The two different media, WA + ARE and PDA, for which fungal and oomycetal volatile production was analyzed, were also used for screening the bacterial response to volatiles. Out of all phenotypes tested (growth, antimicrobial activity, biofilm formation, and motility), motility was the only phenotype affected by the fungal volatiles.

**FIGURE 3:**
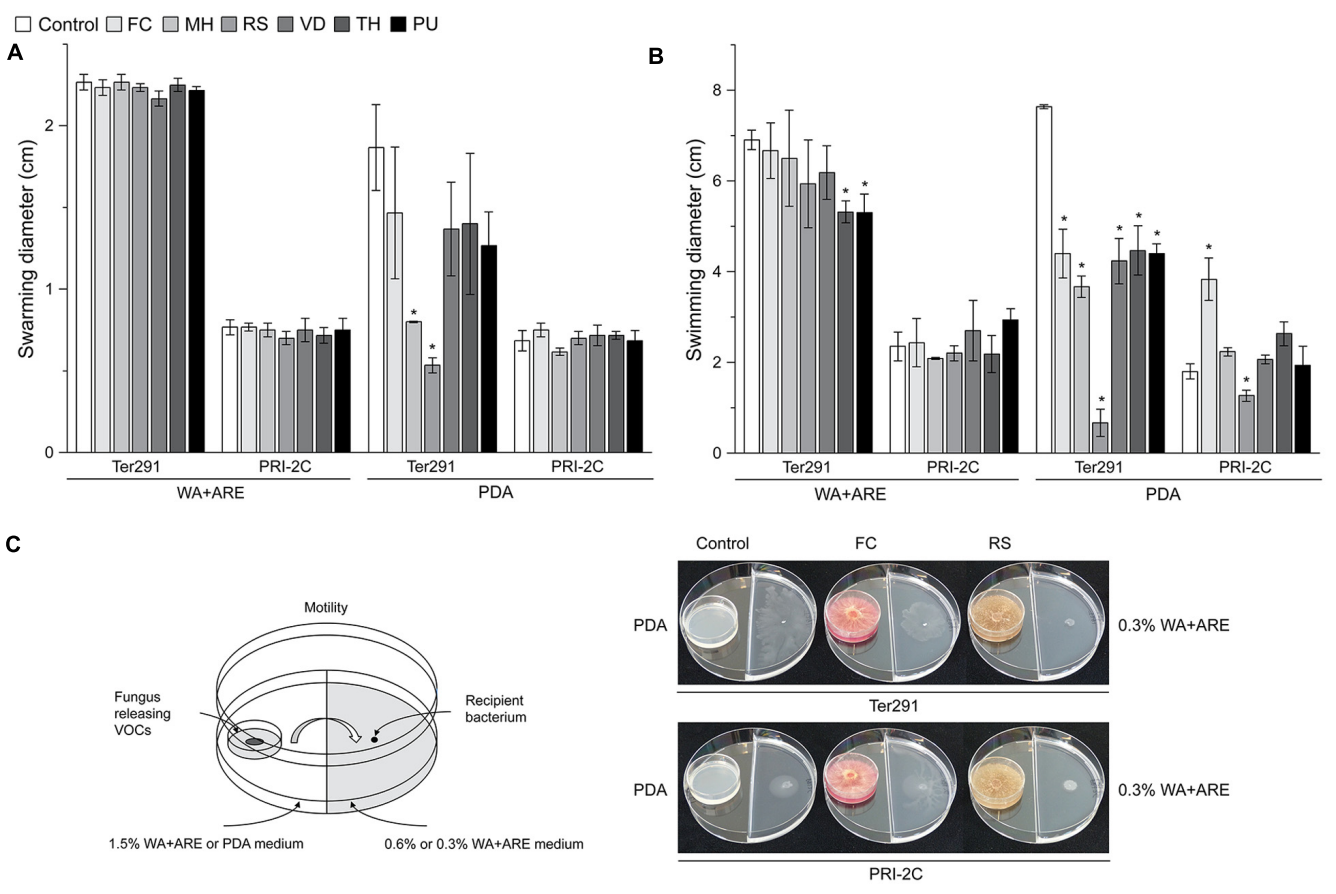
**Effect of fungal and oomycetal volatiles on *Collimonas pratensis* Ter291 and *Serratia plymuthica* PRI-2C swarming motility (0.6% wt/vol agar) and swimming motility (0.3% wt/vol agar) on water agar supplied with artificial root exudates (A) and potato dextrose agar (B).** Setup of experiment and effect of FC and RS volatiles on swimming motility of *C. pratensis* Ter291 and *S. plymuthica* PRI-2C **(C)**. MH, *M. hiemalis*; RS, *R. solani*; PU, *P. ultimum*; VD, *V. dahliae*; FC, *F. culmorum*; TH, *T. harzianum*, Control: media. Five microliters of washed overnight cultures of *C. pratensis* Ter291 and *S. plymuthica* PRI-2C was spotted in the center of a soft agar partitioned Petri dish containing the fungal and oomycetal strains and incubated for 3 days at 20°C. As an indicator of motility the average swimming and swarming diameter (cm) was measured. The error bars represent standard errors of the mean of three independent biological replicates. The asterisks indicate statistically significant (*P* < 0.05) differences relative to the control.

From all bacterial strains screened, *C. pratensis* Ter291 and *S. plymuthica* PRI-2C revealed the strongest and highly reproducible responses in motility upon exposure to the fungal and oomycetal volatiles (**Figure 3** and **Supplementary Table S1**). For the other bacterial strains high variability was observed between replicates (**Supplementary Table S1**). Consequently, we focused on the description of the response in motility of *C. pratensis* Ter291 and *S. plymuthica* PRI-2C.

Overall, swimming motility was more strongly affected than swarming motility and the effect on motility was much more pronounced by fungal and oomycetal volatiles emitted from PDA than from WA + ARE (**Figure 3**). Only *C. pratensis* Ter291 swarming motility was significantly reduced when exposed to volatiles emitted by *M. hiemalis* and *R. solani* on PDA (**Figure 3A**). No significant effect was observed in swarming motility of *C. pratensis* Ter291 and *S. plymuthica* PRI-2C by fungal and oomycetal volatiles emitted from WA + ARE and for *S. plymuthica* PRI-2C by volatiles emitted from PDA (**Figure 3A**).

The swimming motility of *C. pratensis* Ter291 was significantly reduced by volatiles emitted by *T. harzianum* and *P. ultimum* growing on WA + ARE (**Figure 3B**). No such effect was observed for *S. plymuthica* PRI-2C. Volatiles emitted by all fungal and oomycetal strains growing on PDA revealed a significant reduction of the swimming motility of *C. pratensis* Ter291 with a very strong effect observed by *R. solani* (**Figures 3B,C**). For *S. plymuthica* PRI-2C swimming motility was significantly induced when exposed to volatiles produced by *F. culmorum* on PDA whereas the swimming motility was significantly reduced when exposed to volatiles produced by *R. solani* on PDA (**Figures 3B,C**).

Volatiles emitted by *R. solani* growing on PDA reduced swarming as well swimming motility (**Figures 3B,C**). The HCA (**Supplementary Figure S2B**, cluster 1) resulted in a unique cluster for *R. solani*, however, the compounds could not be identified with certainty and thus no correlation between the reduction in motility and the potentially involved compounds could be drawn.

Interestingly, volatiles emitted by one fungus lead to different responses in the two strains. For example *F. culmorum* grown on PDA revealed a reduction of swimming motility of *C. pratensis* Ter291 and an induction in *S. plymuthica* PRI-2C (**Figures 3B,C**). As shown in the HCA, *F. culmorum* is characterized by a unique cluster of volatiles, consisting primarily of terpenes (**Figure 1**).

### Effect of Individual Terpenes on *S. plymuthica* PRI-2C and *C. pratensis* Ter291 Motility

To test whether terpenes may play a role in the observed motility response of *S. plymuthica* PRI-2C and *C. pratensis* Ter291, four terpenes (α-Terpinene, β-Phellandrene, 3-Carene, and Camphene) were selected from the unique *F. culmorum* cluster to be tested individually. These compounds showed a reliable annotation based on their retention indices and mass spectral similarity, with a match score >800, and were commercially (synthetically) available as authentic standards. The identity of the four pure compounds was confirmed by GC-MS by comparing their mass spectra and RI with those found in the *F. culmorum* volatile profile. Their respective mass spectra are given in **Supplementary Figure S3**. A range of different concentrations, previously reported in experiments with microorganisms (Blom et al., 2011; Kim et al., 2013), was used to test their effect on motility of *S. plymuthica* PRI-2C and *C. pratensis* Ter291.

**FIGURE 4:**
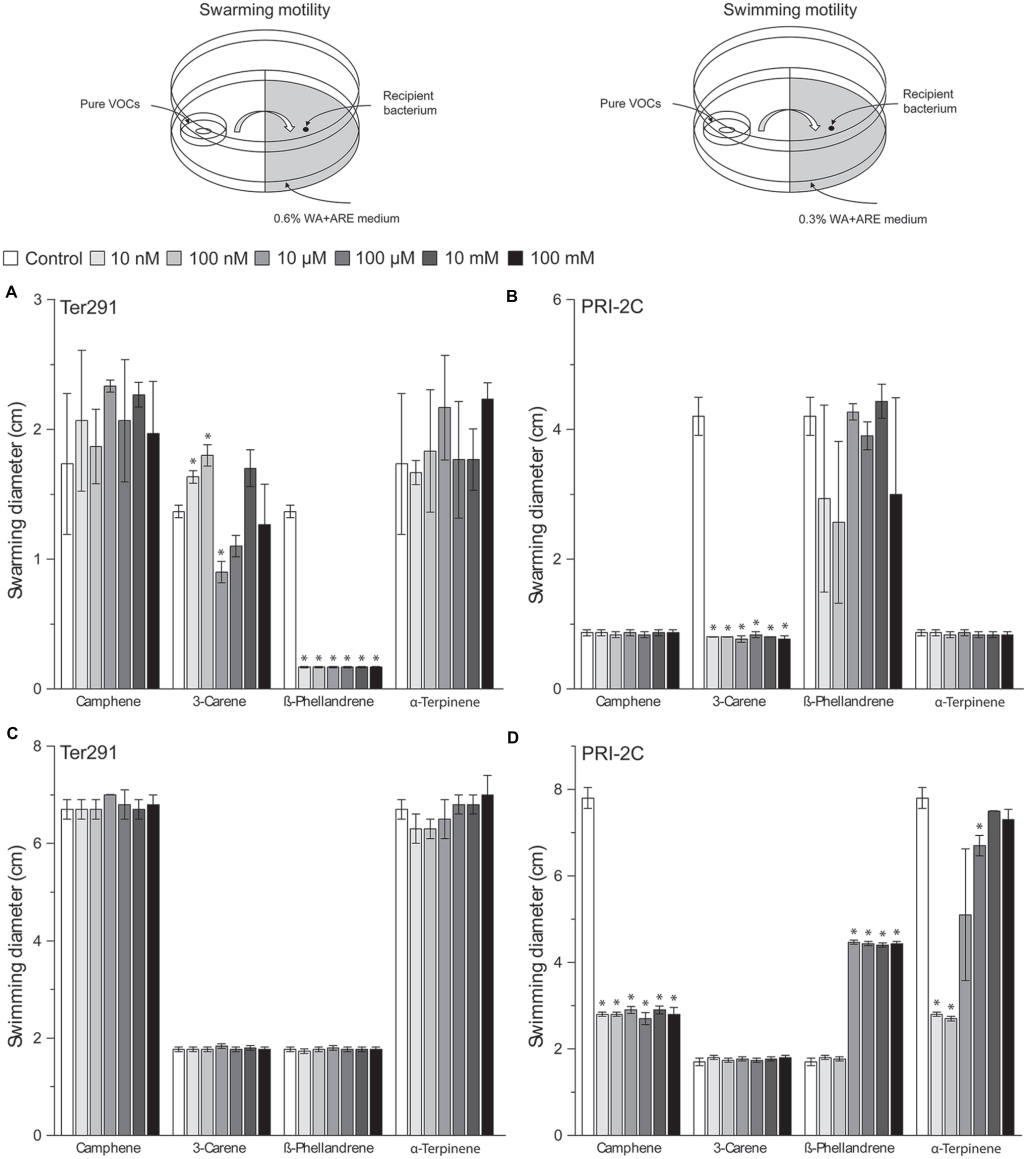
**Setup of experiment and effect of pure volatiles on *Collimonas pratensis* Ter291 and *Serratia plymuthica* PRI-2C swarming motility (0.6% wt/vol agar) (A,B) and swimming motility (0.3% wt/vol agar) (C,D) on water agar supplied with artificial root exudates.** Control: media. Five microliters of washed overnight cultures of *C. pratensis* Ter291 and *S. plymuthica* PRI-2C was spotted in the center of a soft agar partitioned Petri dish containing the pure volatiles and incubated for 3 days at 20°C. As an indicator of motility the average swimming and swarming diameter (cm) was measured. The error bars represent standard errors of the mean of three independent biological replicates. The asterisks indicate statistically significant (*P* < 0.05) differences relative to the control.

The screening showed that *S. plymuthica* PRI-2C was affected in both swarming and swimming motility, while *C. pratensis* Ter291 was only affected in swarming motility (**Figure 4**). For some of the pure compounds, a concentration dependent effect was observed. For example, α-Terpinene affected *S. plymuthica* PRI-2C swimming motility in concentrations of 10 nm, 100 nM, and 100 μM, but no effect was observed with high concentrations (10 mM and 100 mM; **Figure 4D**). ß-Phellandrene induced swimming motility in *S. plymuthica* PRI-2C in concentrations from 10 μM to 100 mM (**Figure 4D**). Interestingly, depending on the concentration, 3-Carene affected the swarming motility in *C. pratensis* Ter291 in different ways. At concentrations of 10 and 100 nM, *C. pratensis* Ter291 swarming motility was significantly increased while being significantly decreased at concentration of 10 μM (**Figure 4A**). Independently of the concentrations applied, ß-Phellandrene significantly reduced swarming motility of *C. pratensis* Ter291 (**Figure 4A**). Likewise, 3-Carene and Camphene lead to a significant decrease in *S. plymuthica* PRI-2C swarming and swimming motility, respectively (**Figures 4B,D**).

## Discussion

Volatile compounds form an important part in the interactions between different soil inhabiting microorganisms (Insam and Seewald, 2010; Wenke et al., 2010; Effmert et al., 2012; Garbeva et al., 2014a). They can have different ecological functions, including inhibition or promotion of other (micro)-organisms (Kai et al., 2007, 2009; Vespermann et al., 2007; Bailly and Weisskopf, 2012; Bitas et al., 2013). However, one important role that has been long overlooked is the ability of volatiles to act as signaling molecules in the communication between different soil microorganisms despite their physico-chemical properties that facilitate diffusion through soil. To date, very little is known about the role of fungal volatiles in fungal–bacterial interactions. Thus, the aim of this study was to investigate the effect of fungal volatiles on bacteria.

Our results added to fill this gap in knowledge by showing that fungal and oomycetal volatiles can play an important role in long distance fungal–bacterial interactions, and can lead to specific phenotypical responses in the interacting partners. Out of all the phenotypical responses considered namely growth, antimicrobial activity, biofilm formation and motility, motility of bacteria, both swimming (individual cells moving in more liquid environments) and swarming (direct, signal-dependent movement powered by rotating flagella), were significantly affected. Fungal and oomycetal volatiles either triggered or suppressed bacterial motility depending on the interacting partner. This finding could, therefore, reflect a potential strategy employed by the fungus to attract mutualistic bacteria toward itself and to repel competitors from common niches by manipulating their motility through volatiles. The composition and abundance of volatiles is affected by the growth stage of the fungal/oomycetal strains and the nutrient conditions. Several independent studies have reported that the volatile profiles of bacteria are also dependent on growth condition and nutrient availability (Korpi et al., 2009; Insam and Seewald, 2010; Blom et al., 2011; Bitas et al., 2013; Garbeva et al., 2014b).

Besides the growth stage- and nutrient condition-dependent changes in the global volatile profile, certain groups of volatiles are emitted in higher amounts by specific individual strains. Terpenes emitted by *F. culmorum* are the most salient example from our study: a nutrient-poor growing condition triggers higher levels of terpene emission at an early growth stage and an even higher emission at a later growth stage; on the contrary, under nutrient-rich conditions, the emission of this volatile cluster was induced only at a later growth stage. This suggests that fungi and oomycetes can invest their carbon resources toward formation of specific blends of volatiles depending on their growth stages and the nutrient availability in their environment. Our findings are in line with those by Korpi et al. (2009), who demonstrated that a lack of certain nutrients leads to terpene emission, suggesting that some volatiles are produced only under nutrient-limited conditions, which is often the case in natural environments. Terpenes represent the biggest and most diverse family of primary and secondary metabolites found in a variety of organisms, among which several fungi (Keller et al., 2005; Gioacchini et al., 2008; Strobel et al., 2011; Muller et al., 2013; Busko et al., 2014). Most studies, however, focused mainly on the detection and chemical characterization of these molecules, while only few addressed the biological function of terpenes. The latter studies indicate that fungal terpenes may be used in defense against competitors (e.g., caryophyllene) or as a signaling molecule (e.g., farnesol; Martins et al., 2007). Our work, based on the screening of fungal and oomycetal strains and using pure terpene compounds, proved that individual terpenes affect the motility of the exposed bacteria.

Several studies showed that fungi have a high sensitivity to volatiles emitted by bacteria leading to reduction and inhibition in spore germination and growth (fungistasis; Garbeva et al., 2011, 2014b; Effmert et al., 2012; Schmidt et al., 2015). The difference in susceptibility between fungi and oomycetes may be due to the structure of their cell wall (Schmidt et al., 2015). It was recently proposed that bacteriostasis (inability of bacteria to multiply in soil; Ho and Ko, 1982; Effmert et al., 2012) might also involve volatile compounds. However, within this study we did not observed effect on bacterial growth by fungal volatiles. In contrast to fungi, bacteria seem to be more resistant to volatiles. It has been speculated that variations in sensitivity of bacteria to volatiles may possibly be mediated by an ATP-dependent eﬄux mechanism, which has been investigated for several terpene compounds against *Pseudomonas aeruginosa* (Cox and Markham, 2007) as well as the ability of volatiles to disintegrate the outer membrane (Longbottom et al., 2004). These findings may indicate that bacteria are more resistant to volatiles emitted by fungi and oomycetes.

The identity of volatile molecules is an important basis for understanding their ecological roles. However, it is a challenging task to unambiguously identify the high number of compounds detected, just as it is to set the right ranges of concentrations that are representative of the natural condition during screenings with pure compounds. The technology used in this study does not allow measuring the actual concentration of the volatile compounds produced by the strains. Thus, when testing the effect of pure compounds we adopted a range of concentrations known to be relevant in such assays (Blom et al., 2011; Kim et al., 2013). Interestingly, some of the pure compounds showed a dose-dependent effect on the motility. This suggests that by regulating the emission of volatiles, fungi might be able to influence bacterial responses in different ways. For instance, the emission of volatiles in lower concentrations might attract the bacterium to move toward the fungus, while volatiles emitted in higher concentrations might be toxic and thus repel the bacteria away from the fungus. For example, bacteria from the genus *Collimonas*, used in this study, were previously shown to colonize and grow on living fungal hyphae (a phenomenon called mycophagy; de Boer, 2004), implying that volatiles might play a role as long-distance signals for attracting such mycophagous bacteria.

## Conclusion

Bacteria can sense fungal and oomycetal volatiles and respond with changes in motility. This response was dependent on the volatile blend emitted by the organisms, which was influenced by the nutrient conditions and, for some strains, by their growth stage. Several identified volatile terpenes were shown to affect motility. To better understand how bacteria perceive fungal volatiles on a cellular level, a valuable insight could stem from future studies involving transcriptomics and proteomics tools.

## Conflict of Interest Statement

The authors declare that the research was conducted in the absence of any commercial or financial relationships that could be construed as a potential conflict of interest.

## Supplementary material

TABLE S1 **Effect of fungal and oomycetal volatiles on bacterial phenotypes (growth, antimicrobial activity, biofilm formation and motility) on WA+ARE and PDA**.

FIGURE S1 **Variations of assays in double plate-within-a-plate system used to test the effect of fungal volatile compounds on bacterial growth and antimicrobial activity (A), motility (B) and biofilm formation (C) as described in section “Materials and Methods**.”

FIGURE S2 **Hierarchical cluster analysis (HCA) of fungal and oomycetal strains based on volatiles that were significantly different (*P* < 0.05 with Bonferroni correction) in abundance between at least two strains when grown on water agar supplied with artificial root exudates (A) and on potato dextrose agar (B) at day 3 (early growth stage) and day 6 (late growth stage)**. MH, *Mucor hiemalis*; RS, *Rhizoctonia solani*; PU, *Pythium ultimum*; VD, *Verticillium dahliae*; FC, *Fusarium culmorum*; TH, *Trichoderma harzianum*; C, Control (media). The color code below the figure indicates the abundance of the volatiles, which is log2-transformed and scaled by Mean/SD. The boxes indicated in different colors discern the eight distinct clusters that determine the spatial separation of the samples in the PCA.

FIGURE S3 **Comparison of mass spectra of the pure volatile compounds with those found in *F. culmorum***.

## References

[B1] Bailly A. Weisskopf L. (2012). The modulating effect of bacterial volatiles on plant growth: current knowledge and future challenges. *Plant Signal. Behav.* 7 79–85. 10.4161/psb.7.1.18418 22301973 PMC3357376

[B2] Bitas V. Kim H. S. Bennett J. W. Kang S. (2013). Sniffing on microbes: diverse roles of microbial volatile organic compounds in plant health. *Mol. Plant Microbe Interact.* 26 835–843. 10.1094/MPMI-10-12-0249-CR 23581824

[B3] Blom D. Fabbri C. Connor E. C. Schiestl F. P. Klauser D. R. Boller T. (2011). Production of plant growth modulating volatiles is widespread among rhizosphere bacteria and strongly depends on culture conditions. *Environ. Microbiol.* 13 3047–3058. 10.1111/j.1462-2920.2011.02582.x 21933319

[B4] Busko M. Kulik T. Ostrowska A. Goral T. Perkowski J. (2014). Quantitative volatile compound profiles in fungal cultures of three different *Fusarium graminearum* chemotypes. *FEMS Microbiol. Lett.* 359 85–93. 10.1111/1574-6968.12569 25132145

[B5] Cox S. D. Markham J. L. (2007). Susceptibility and intrinsic tolerance of *Pseudomonas aeruginosa* to selected plant volatile compounds. *J. Appl. Microbiol.* 103 930–936. 10.1111/j.1365-2672.2007.03353.x 17897196

[B6] de Boer W. (2004). *Collimonas fungivorans* gen. nov., sp. nov., a chitinolytic soil bacterium with the ability to grow on living fungal hyphae. *Int. J. Syst. Evol. Microbiol.* 54 857–864. 10.1099/ijs.0.02920-0 15143036

[B7] De Boer W. Klein Gunnewiek P. J. A. Lafeber P. Janse J. D. Spit B. E. Woldendorp J. W. (1998). Anti-fungal properties of chitinolytic dune soil bacteria. *Soil Biol. Biochem.* 30 193–203. 10.1016/S0038-0717(97)00100-4

[B8] de Boer W. Wagenaar A. M. Klein Gunnewiek P. J. Van Veen J. A. (2007). In vitro suppression of fungi caused by combinations of apparently non-antagonistic soil bacteria. *FEMS Microbiol. Ecol.* 59 177–185. 10.1111/j.1574-6941.2006.00197.x 17233750

[B9] de Bruijn I. Raaijmakers J. M. (2009). Regulation of cyclic lipopeptide biosynthesis in *Pseudomonas fluorescens* by the ClpP protease. *J. Bacteriol.* 191 1910–1923. 10.1128/JB.01558-08 19114474 PMC2648375

[B10] De Ridder-Duine A. S. Kowalchuk G. A. Klein Gunnewiek P. J. A. Smant W. Van Veen J. A. De Boer W. (2005). Rhizosphere bacterial community composition in natural stands of *Carex arenaria* (sand sedge) is determined by bulk soil community composition. *Soil Biol. Biochem.* 37 349–357. 10.1016/j.soilbio.2004.08.005

[B11] Effmert U. Kalderas J. Warnke R. Piechulla B. (2012). Volatile mediated interactions between bacteria and fungi in the soil. *J. Chem. Ecol.* 38 665–703. 10.1007/s10886-012-0135-5 22653567

[B12] Fiddaman P. J. Rossall S. (1993). The production of antifungal volatiles by *Bacillus subtilis*. *J. Appl. Bacteriol.* 74 119–126. 10.1111/j.1365-2672.1993.tb03004.x 8444640

[B13] Frey-Klett P. Burlinson P. Deveau A. Barret M. Tarkka M. Sarniguet A. (2011). Bacterial-fungal interactions: hyphens between agricultural, clinical, environmental, and food microbiologists. *Microbiol. Mol. Biol. Rev.* 75 583–609. 10.1128/MMBR.00020-11 22126995 PMC3232736

[B14] Garbeva P. de Boer W. (2009). Inter-specific interactions between carbon-limited soil bacteria affect behavior and gene expression. *Microb. Ecol.* 58 36–46. 10.1007/s00248-009-9502-3 19267150

[B15] Garbeva P. Hol W. H. G. Termorshuizen A. J. Kowalchuk G. A. De Boer W. (2011). Fungistasis and general soil biostasis – A new synthesis. *Soil Biol. Biochem.* 43 469–477. 10.1016/j.soilbio.2010.11.020

[B16] Garbeva P. Hordijk C. Gerards S. De Boer W. (2014a). Volatile-mediated interactions between phylogenetically different soil bacteria. *Front. Microbiol.* 5:289 10.3389/fmicb.2014.00289 PMC4052926 24966854

[B17] Garbeva P. Hordijk C. Gerards S. De Boer W. (2014b). Volatiles produced by the mycophagous soil bacterium *Collimonas.* *FEMS Microbiol. Ecol.* 87 639–649. 10.1111/1574-6941.12252 24329759

[B18] Garbeva P. Voesenek K. Elsas J. D. V. (2004). Quantitative detection and diversity of the pyrrolnitrin biosynthetic locus in soil under different treatments. *Soil Biol. Biochem.* 36 1453–1463. 10.1016/j.soilbio.2004.03.009

[B19] Gioacchini A. M. Menotta M. Guescini M. Saltarelli R. Ceccaroli P. Amicucci A. (2008). Geographical traceability of Italian white truﬄe (*Tuber magnatum* Pico) by the analysis of volatile organic compounds. *Rapid Commun. Mass Spectr.* 22 3147–3153. 10.1002/rcm.3714 18798200

[B20] Haq I. U. Zhang M. Yang P. Van Elsas J. D. (2014). The interactions of bacteria with fungi in soil: emerging concepts. *Adv. Appl. Microbiol.* 89 185–215. 10.1016/B978-0-12-800259-9.00005-6 25131403

[B21] Ho W. C. Ko W. H. (1982). Characteristics of soil microbiostasis. *Soil Biol. Biochem.* 14 589–593. 10.1016/0038-0717(82)90092-X

[B22] Huang X. (2014). Horizontal transfer generates genetic variation in an asexual pathogen. *PeerJ* 2:e650 10.7717/peerj.650 PMC4217194 25374789

[B23] Hung R. Lee S. Bennett J. W. (2015). Fungal volatile organic compounds and their role in ecosystems. *Appl. Microbiol. Biotechnol.* 99 3395–3405. 10.1007/s00253-015-6494-4 25773975

[B24] Insam H. Seewald M. S. A. (2010). Volatile organic compounds (VOCs) in soils. *Biol. Fertil. Soils* 46 199–213. 10.1007/s00374-010-0442-3

[B25] Kai M. Effmert U. Berg G. Piechulla B. (2007). Volatiles of bacterial antagonists inhibit mycelial growth of the plant pathogen *Rhizoctonia solani*. *Arch. Microbiol.* 187 351–360. 10.1007/s00203-006-0199-0 17180381

[B26] Kai M. Haustein M. Molina F. Petri A. Scholz B. Piechulla B. (2009). Bacterial volatiles and their action potential. *Appl. Microbiol. Biotechnol.* 81 1001–1012. 10.1007/s00253-008-1760-3 19020812

[B27] Keller N. P. Turner G. Bennett J. W. (2005). Fungal secondary metabolism - From biochemistry to genomics. *Nat. Rev. Microbiol.* 3 937–947. 10.1038/Nrmicro1286 16322742

[B28] Kim K. S. Lee S. Ryu C. M. (2013). Interspecific bacterial sensing through airborne signals modulates locomotion and drug resistance. *Nat. Commun.* 4:1809 10.1038/ncomms2789 23651997

[B29] Korpi A. Jarnberg J. Pasanen A. L. (2009). Microbial volatile organic compounds. *Crit. Rev. Toxicol* 39 139–193. 10.1080/10408440802291497 19204852

[B30] Lee S. Hung R. Yap M. Bennett J. W. (2015). Age matters: the effects of volatile organic compounds emitted by *Trichoderma atroviride* on plant growth. *Arch. Microbiol.* 197 723–727. 10.1007/s00203-015-1104-5 25771960

[B31] Leveau J. H. Uroz S. De Boer W. (2010). The bacterial genus Collimonas: mycophagy, weathering and other adaptive solutions to life in oligotrophic soil environments. *Environ. Microbiol.* 12 281–292. 10.1111/j.1462-2920.2009.02010.x 19638176

[B32] Lommen A. (2009). MetAlign: interface-driven, versatile metabolomics tool for hyphenated full-scan mass spectrometry data preprocessing. *Anal. Chem.* 81 3079–3086. 10.1021/ac900036d 19301908

[B33] Longbottom C. J. Carson C. F. Hammer K. A. Mee B. J. Riley T. V. (2004). Tolerance of *Pseudomonas aeruginosa* to *Melaleuca alternifolia* (tea tree) oil is associated with the outer membrane and energy-dependent cellular processes. *J. Antimicrob. Chemother.* 54 386–392. 10.1093/jac/dkh359 15254026

[B34] Martins M. Henriques M. Azeredo J. Rocha S. M. Coimbra M. A. Oliveira R. (2007). Morphogenesis Control in *Candida albicans* and *Candida dubliniensis* through Signaling Molecules Produced by Planktonic and Biofilm Cells. *Eukaryot. Cell* 6 2429–2436. 10.1128/EC.00252-07 17981993 PMC2168255

[B35] Mela F. Fritsche K. De Boer W. Van Veen J. A. De Graaff L. H. Van Den Berg M. (2011). Dual transcriptional profiling of a bacterial/fungal confrontation: *Collimonas fungivorans* versus *Aspergillus niger*. *ISME J.* 5 1494–1504. 10.1038/ismej.2011.29 21614084 PMC3160687

[B36] Morath S. U. Hung R. Bennett J. W. (2012). Fungal volatile organic compounds: a review with emphasis on their biotechnological potential. *Fungal Biol. Rev.* 26 73–83. 10.1016/j.fbr.2012.07.001

[B37] Muller A. Faubert P. Hagen M. Zu Castell W. Polle A. Schnitzler J. P. (2013). Volatile profiles of fungi–chemotyping of species and ecological functions. *Fungal Genet. Biol.* 54 25–33. 10.1016/j.fgb.2013.02.005 23474123

[B38] O’Toole G. A. Pratt L. A. Watnick P. I. Newman D. K. Weaver V. B. Kolter R. (1999). “[6] Genetic approaches to study of biofilms,” in *Methods in Enzymology*, ed. Ron J. D. (Waltham, MA: Academic Press), 91–109. 10.1016/s0076-6879(99)10008-9 10547784

[B39] Piechulla B. Degenhardt J. (2014). The emerging importance of microbial volatile organic compounds. *Plant Cell Environ.* 37 811–812. 10.1111/pce.12254 24329873

[B40] Romoli R. Papaleo M. C. De Pascale D. Tutino M. L. Michaud L. Logiudice A. (2014). GC–MS volatolomic approach to study the antimicrobial activity of the antarctic bacterium Pseudoalteromonas sp. *TB*41. *Metabolomics* 10 42–51. 10.1007/s11306-013-0549-2

[B41] Ryan R. P. Dow J. M. (2008). Diffusible signals and interspecies communication in bacteria. *Microbiology* 154 1845–1858. 10.1099/mic.0.2008/017871-0 18599814

[B42] Schmidt R. Cordovez V. De Boer W. Raaijmakers J. Garbeva P. (2015). Volatile affairs in microbial interactions. *ISME J.* 2329–2335. 10.1038/ismej.2015.42 26023873 PMC4611499

[B43] Schulz S. Dickschat J. S. (2007). Bacterial volatiles: the smell of small organisms. *Nat. Prod. Rep.* 24 814–842. 10.1039/b507392h 17653361

[B44] Strobel G. Singh S. K. Riyaz-Ul-Hassan S. Mitchell A. M. Geary B. Sears J. (2011). An endophytic/pathogenic Phoma sp. from creosote bush producing biologically active volatile compounds having fuel potential. *FEMS Microbiol. Lett.* 320 87–94. 10.1111/j.1574-6968.2011.02297.x 21535100

[B45] Sumner L. W. Amberg A. Barrett D. Beale M. H. Beger R. Daykin C. A. (2007). Proposed minimum reporting standards for chemical analysis Chemical Analysis Working Group (CAWG) Metabolomics Standards Initiative (MSI). *Metabolomics* 3 211–221. 10.1007/s11306-007-0082-2 24039616 PMC3772505

[B46] Tikunov Y. M. Laptenok S. Hall R. D. Bovy A. De Vos R. C. (2012). MSClust: a tool for unsupervised mass spectra extraction of chromatography-mass spectrometry ion-wise aligned data. *Metabolomics* 8 714–718. 10.1007/s11306-011-0368-2 22833709 PMC3397229

[B47] Tyc O. Van Den Berg M. Gerards S. Van Veen J. De Boer W. Raaijmakers J. M. (2014). Frequency of interaction-mediated triggering of antibiotic production among soil bacteria. *Front. Microbiol.* 5:567 10.3389/fmicb.2014.00567 PMC4211544 25389421

[B48] Vespermann A. Kai M. Piechulla B. (2007). Rhizobacterial volatiles affect the growth of fungi and *Arabidopsis thaliana*. *Appl. Environ. Microbiol.* 73 5639–5641. 10.1128/AEM.01078-07 17601806 PMC2042089

[B49] Weise T. Kai M. Gummesson A. Troeger A. Von Reuss S. Piepenborn S. (2012). Volatile organic compounds produced by the phytopathogenic bacterium *Xanthomonas campestris* pv. vesicatoria 85-10. *Beilstein J. Org. Chem.* 8 579–596. 10.3762/bjoc.8.65 22563356 PMC3343284

[B50] Wenke K. Kai M. Piechulla B. (2010). Belowground volatiles facilitate interactions between plant roots and soil organisms. *Planta* 231 499–506. 10.1007/s00425-009-1076-2 20012987

[B51] Wheatley R. E. (2002). The consequences of volatile organic compound mediated bacterial and fungal interactions. *Antonie Van Leeuwenhoek* 81 357–364. 10.1023/A:1020592802234 12448734

