# Supplementary material for: Microbial Small Talk: Volatiles in Fungal–Bacterial Interactions
Source: Front Microbiol. 2016 Jan 5;6:1495. doi: 10.3389/fmicb.2015.01495 (PMC4700264; doi:10.3389/fmicb.2015.01495)
Supplement: TABLE S1 — Effect of fungal and oomycetal volatiles on bacterial phenotypes (growth, antimicrobial activity, biofilm formation and motility) on WA+ARE and PDA. [file Table_1.DOCX]

**Table S1.** Effect of fungal and oomycetal volatiles on bacterial phenotypes (growth, antimicrobial activity, biofilm formation and motility) on WA+ARE and PDA.

| **WA+ARE** |  | | | | | | |
| --- | --- | --- | --- | --- | --- | --- | --- |
|  | ***Collimonas fungivorans* Ter6** | | | | | | |
|  | Growth | Antimicrobial activity | | | Biofilm formation | Motility | |
|  |  | *E.coli* ET12567 | *E.coli* WA321 | *S.aureus* |  | 0.3% | 0.6% |
| *Mucor hiemalis* | = | = | = | = | = | = | ↘ |
| *Rhizoctonia solani* | = | = | = | = | = | ↘ | ↗ |
| *Fusarium culmorum* | = | = | = | = | = | = | ↗ |
| *Verticillium dahliae* | = | = | = | = | = | = | = |
| *Trichoderma harzianum* | = | = | = | = | = | ↘ | ↗ |
| *Pythium ultimum* | = | = | = | = | = | ↘ | = |
|  | ***Collimonas fungivorans* Ter331** | | | | | | |
|  | Growth | Antimicrobial activity | | | Biofilm formation | Motility | |
|  |  | *E.coli* ET12567 | *E.coli* WA321 | *S.aureus* |  | 0.3% | 0.6% |
| *Mucor hiemalis* | = | = | = | = | = | = | = |
| *Rhizoctonia solani* | = | = | = | = | = | = | = |
| *Fusarium culmorum* | = | = | = | = | = | = | = |
| *Verticillium dahliae* | = | = | = | = | = | = | = |
| *Trichoderma harzianum* | = | = | = | = | = | NR | NR |
| *Pythium ultimum* | = | = | = | = | = | NR | NR |
|  | ***Collimonas pratensis* Ter91** |  |  |  |  |  |  |
|  | Growth | Antimicrobial activity | | | Biofilm formation | Motility | |
|  |  | *E.coli* ET12567 | *E.coli* WA321 | *S.aureus* |  | 0.3% | 0.6% |
| *Mucor hiemalis* | = | = | = | = | = | = | = |
| *Rhizoctonia solani* | = | = | = | = | = | ↘ | ↗ |
| *Fusarium culmorum* | = | = | = | = | = | = | = |
| *Verticillium dahliae* | = | = | = | = | = | ↗ | = |
| *Trichoderma harzianum* | = | = | = | = | = | = | ↗ |
| *Pythium ultimum* | = | = | = | = | = | ↘ | ↘ |
|  | ***Collimonas pratensis* Ter291** |  |  |  |  |  |  |
|  | Growth | Antimicrobial activity | | | Biofilm formation | Motility | |
|  |  | *E.coli* ET12567 | *E.coli* WA321 | *S.aureus* |  | 0.3% | 0.6% |
| *Mucor hiemalis* | = | = | = | = | = | = | = |
| *Rhizoctonia solani* | = | = | = | = | = | ↘ | = |
| *Fusarium culmorum* | = | = | = | = | = | ↘ | = |
| *Verticillium dahliae* | = | = | = | = | = | = | = |
| *Trichoderma harzianum* | = | = | = | = | = | ↘ | ↗ |
| *Pythium ultimum* | = | = | = | = | = | ↗ | = |
|  | ***Collimonas arenae* Ter282** |  |  |  |  |  |  |
|  | Growth | Antimicrobial activity | | | Biofilm formation | Motility | |
|  |  | *E.coli* ET12567 | *E.coli* WA321 | *S.aureus* |  | 0.3% | 0.6% |
| *Mucor hiemalis* | = | = | = | = | = | ↘ | = |
| *Rhizoctonia solani* | = | = | = | = | = | ↗ | = |
| *Fusarium culmorum* | = | = | = | = | = | = | = |
| *Verticillium dahliae* | = | = | = | = | = | ↘ | ↗ |
| *Trichoderma harzianum* | = | = | = | = | = | = | = |
| *Pythium ultimum* | = | = | = | = | = | ↘ | ↘ |
|  | ***Collimonas arenae* Ter10** |  |  |  |  |  |  |
|  | Growth | Antimicrobial activity | | | Biofilm formation | Motility | |
|  |  | *E.coli* ET12567 | *E.coli* WA321 | *S.aureus* |  | 0.3% | 0.6% |
| *Mucor hiemalis* | = | = | = | = | = | = | = |
| *Rhizoctonia solani* | = | = | = | = | = | ↗ | = |
| *Fusarium culmorum* | = | = | = | = | = | = | = |
| *Verticillium dahliae* | = | = | = | = | = | = | = |
| *Trichoderma harzianum* | = | = | = | = | = | ↘ | = |
| *Pythium ultimum* | = | = | = | = | = | ↘ | = |
|  | ***Burkholderia sp.* AD24** |  |  |  |  |  |  |
|  | Growth | Antimicrobial activity | | | Biofilm formation | Motility | |
|  |  | *E.coli* ET12567 | *E.coli* WA321 | *S.aureus* |  | 0.3% | 0.6% |
| *Mucor hiemalis* | = | = | = | = | = | ↗ | = |
| *Rhizoctonia solani* | = | = | = | = | = | = | = |
| *Fusarium culmorum* | = | = | = | = | = | ↗ | = |
| *Verticillium dahliae* | = | = | = | = | = | ↘ | = |
| *Trichoderma harzianum* | = | = | = | = | = | NR | NR |
| *Pythium ultimum* | = | = | = | = | = | NR | NR |
|  | ***Serratia plymutica* PRI-2C** |  |  |  |  |  |  |
|  | Growth | Antimicrobial activity | | | Biofilm formation | Motility | |
|  |  | *E.coli* ET12567 | *E.coli* WA321 | *S.aureus* |  | 0.3% | 0.6% |
| *Mucor hiemalis* | = | = | = | = | = | ↗ | = |
| *Rhizoctonia solani* | = | = | = | = | = | = | ↗ |
| *Fusarium culmorum* | = | = | = | = | = | ↗ | ↗ |
| *Verticillium dahliae* | = | = | = | = | = | = | = |
| *Trichoderma harzianum* | = | = | = | = | = | NR | NR |
| *Pythium ultimum* | = | = | = | = | = | NR | NR |
|  | ***Paenibacillus sp.* AD87** |  |  |  |  |  |  |
|  | Growth | Antimicrobial activity | | | Biofilm formation | Motility | |
|  |  | *E.coli* ET12567 | *E.coli* WA321 | *S.aureus* |  | 0.3% | 0.6% |
| *Mucor hiemalis* | = | = | = | = | = | ↘ | = |
| *Rhizoctonia solani* | = | = | = | = | = | ↘ | = |
| *Fusarium culmorum* | = | = | = | = | = | ↘ | = |
| *Verticillium dahliae* | = | = | = | = | = | ↗ | = |
| *Trichoderma harzianum* | = | = | = | = | = | NR | NR |
| *Pythium ultimum* | = | = | = | = | = | NR | NR |
|  | ***Pedobacter sp.* V48** |  |  |  |  |  |  |
|  | Growth | Antimicrobial activity | | | Biofilm formation | Motility | |
|  |  | *E.coli* ET12567 | *E.coli* WA321 | *S.aureus* |  | 0.3% | 0.6% |
| *Mucor hiemalis* | = | = | = | = | = | = | = |
| *Rhizoctonia solani* | = | = | = | = | = | = | ↘ |
| *Fusarium culmorum* | = | = | = | = | = | = | ↘ |
| *Verticillium dahliae* | = | = | = | = | = | = | ↘ |
| *Trichoderma harzianum* | = | = | = | = | = | NR | NR |
| *Pythium ultimum* | = | = | = | = | = | NR | NR |

| **PDA** |  | | | | | | |
| --- | --- | --- | --- | --- | --- | --- | --- |
|  | ***Collimonas fungivorans* Ter6** | | | | | | |
|  | Growth | Antimicrobial activity | | | Biofilm formation | Motility | |
|  |  | *E.coli* ET12567 | *E.coli* WA321 | *S.aureus* |  | 0.3% | 0.6% |
| *Mucor hiemalis* | = | = | = | = | = | NR | NR |
| *Rhizoctonia solani* | = | = | = | = | = | NR | NR |
| *Fusarium culmorum* | = | = | = | = | = | NR | NR |
| *Verticillium dahliae* | = | = | = | = | = | NR | NR |
| *Trichoderma harzianum* | = | = | = | = | = | NR | NR |
| *Pythium ultimum* | = | = | = | = | = | NR | NR |
|  | ***Collimonas fungivorans* Ter331** | | | | | | |
|  | Growth | Antimicrobial activity | | | Biofilm formation | Motility | |
|  |  | *E.coli* ET12567 | *E.coli* WA321 | *S.aureus* |  | 0.3% | 0.6% |
| *Mucor hiemalis* | = | = | = | = | = | ↗ | ↘ |
| *Rhizoctonia solani* | = | = | = | = | = | ↘ | = |
| *Fusarium culmorum* | = | = | = | = | = | = | = |
| *Verticillium dahliae* | = | = | = | = | = | ↘ | ↘ |
| *Trichoderma harzianum* | = | = | = | = | = | NR | NR |
| *Pythium ultimum* | = | = | = | = | = | NR | NR |
|  | ***Collimonas pratensis* Ter91** |  |  |  |  |  |  |
|  | Growth | Antimicrobial activity | | | Biofilm formation | Motility | |
|  |  | *E.coli* ET12567 | *E.coli* WA321 | *S.aureus* |  | 0.3% | 0.6% |
| *Mucor hiemalis* | = | = | = | = | = | NR | NR |
| *Rhizoctonia solani* | = | = | = | = | = | NR | NR |
| *Fusarium culmorum* | = | = | = | = | = | NR | NR |
| *Verticillium dahliae* | = | = | = | = | = | NR | NR |
| *Trichoderma harzianum* | = | = | = | = | = | NR | NR |
| *Pythium ultimum* | = | = | = | = | = | NR | NR |
|  | ***Collimonas pratensis* Ter291** |  |  |  |  |  |  |
|  | Growth | Antimicrobial activity | | | Biofilm formation | Motility | |
|  |  | *E.coli* ET12567 | *E.coli* WA321 | *S.aureus* |  | 0.3% | 0.6% |
| *Mucor hiemalis* | = | = | = | = | = | ↘ | ↘ |
| *Rhizoctonia solani* | = | = | = | = | = | ↘ | ↘ |
| *Fusarium culmorum* | = | = | = | = | = | ↘ | = |
| *Verticillium dahliae* | = | = | = | = | = | ↘ | = |
| *Trichoderma harzianum* | = | = | = | = | = | ↘ | = |
| *Pythium ultimum* | = | = | = | = | = | ↘ | = |
|  | ***Collimonas arenae* Ter282** |  |  |  |  |  |  |
|  | Growth | Antimicrobial activity | | | Biofilm formation | Motility | |
|  |  | *E.coli* ET12567 | *E.coli* WA321 | *S.aureus* |  | 0.3% | 0.6% |
| *Mucor hiemalis* | = | = | = | = | = | NR | NR |
| *Rhizoctonia solani* | = | = | = | = | = | NR | NR |
| *Fusarium culmorum* | = | = | = | = | = | NR | NR |
| *Verticillium dahliae* | = | = | = | = | = | NR | NR |
| *Trichoderma harzianum* | = | = | = | = | = | NR | NR |
| *Pythium ultimum* | = | = | = | = | = | NR | NR |
|  | ***Collimonas arenae* Ter10** |  |  |  |  |  |  |
|  | Growth | Antimicrobial activity | | | Biofilm formation | Motility | |
|  |  | *E.coli* ET12567 | *E.coli* WA321 | *S.aureus* |  | 0.3% | 0.6% |
| *Mucor hiemalis* | = | = | = | = | = | NR | NR |
| *Rhizoctonia solani* | = | = | = | = | = | NR | NR |
| *Fusarium culmorum* | = | = | = | = | = | NR | NR |
| *Verticillium dahliae* | = | = | = | = | = | NR | NR |
| *Trichoderma harzianum* | = | = | = | = | = | NR | NR |
| *Pythium ultimum* | = | = | = | = | = | NR | NR |
|  | ***Burkholderia sp.* AD24** |  |  |  |  |  |  |
|  | Growth | Antimicrobial activity | | | Biofilm formation | Motility | |
|  |  | *E.coli* ET12567 | *E.coli* WA321 | *S.aureus* |  | 0.3% | 0.6% |
| *Mucor hiemalis* | = | = | = | = | = | ↗ | = |
| *Rhizoctonia solani* | = | = | = | = | = | ↘ | ↗ |
| *Fusarium culmorum* | = | = | = | = | = | = | ↗ |
| *Verticillium dahliae* | = | = | = | = | = | ↗ | ↗ |
| *Trichoderma harzianum* | = | = | = | = | = | NR | NR |
| *Pythium ultimum* | = | = | = | = | = | NR | NR |
|  | ***Serratia plymutica* PRI-2C** |  |  |  |  |  |  |
|  | Growth | Antimicrobial activity | | | Biofilm formation | Motility | |
|  |  | *E.coli* ET12567 | *E.coli* WA321 | *S.aureus* |  | 0.3% | 0.6% |
| *Mucor hiemalis* | = | = | = | = | = | = | = |
| *Rhizoctonia solani* | = | = | = | = | = | ↘ | = |
| *Fusarium culmorum* | = | = | = | = | = | ↗ | = |
| *Verticillium dahliae* | = | = | = | = | = | = | = |
| *Trichoderma harzianum* | = | = | = | = | = | = | = |
| *Pythium ultimum* | = | = | = | = | = | = | = |
|  | ***Paenibacillus sp.* AD87** |  |  |  |  |  |  |
|  | Growth | Antimicrobial activity | | | Biofilm formation | Motility | |
|  |  | *E.coli* ET12567 | *E.coli* WA321 | *S.aureus* |  | 0.3% | 0.6% |
| *Mucor hiemalis* | = | = | = | = | = | = | = |
| *Rhizoctonia solani* | = | = | = | = | = | = | = |
| *Fusarium culmorum* | = | = | = | = | = | = | = |
| *Verticillium dahliae* | = | = | = | = | = | = | = |
| *Trichoderma harzianum* | = | = | = | = | = | NR | NR |
| *Pythium ultimum* | = | = | = | = | = | NR | NR |
|  | ***Pedobacter sp.* V48** |  |  |  |  |  |  |
|  | Growth | Antimicrobial activity | | | Biofilm formation | Motility | |
|  |  | *E.coli* ET12567 | *E.coli* WA321 | *S.aureus* |  | 0.3% | 0.6% |
| *Mucor hiemalis* | = | = | = | = | = | ↗ | ↘ |
| *Rhizoctonia solani* | = | = | = | = | = | ↗ | = |
| *Fusarium culmorum* | = | = | = | = | = | ↗ | ↘ |
| *Verticillium dahliae* | = | = | = | = | = | ↗ | ↘ |
| *Trichoderma harzianum* | = | = | = | = | = | NR | NR |
| *Pythium ultimum* | = | = | = | = | = | NR | NR |

=, not affected; ↘, inhibition; ↗, stimulation; NR, not reproducible
