# Supplementary figures and images for: Microbial Small Talk: Volatiles in Fungal–Bacterial Interactions
Source: Front Microbiol. 2016 Jan 5;6:1495. doi: 10.3389/fmicb.2015.01495 (PMC4700264; doi:10.3389/fmicb.2015.01495)

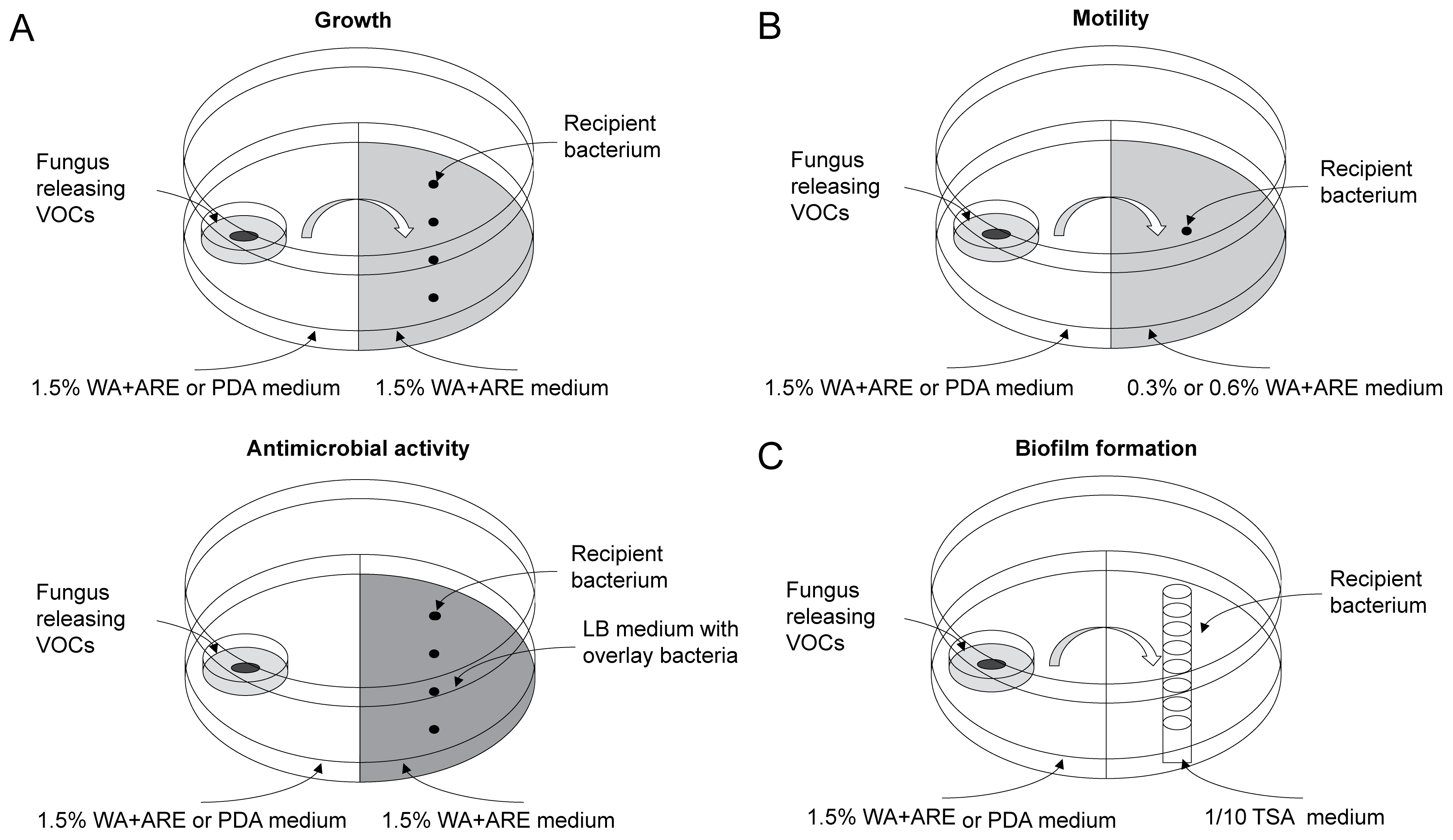

Supplement: FIGURE S1 — Variations of assays in double plate-within-a-plate system used to test the effect of fungal volatile compounds on bacterial growth and antimicrobial activity (A), motility (B) and biofilm formation (C) as described in section “Materials and Methods.” [file Image_1.TIF]

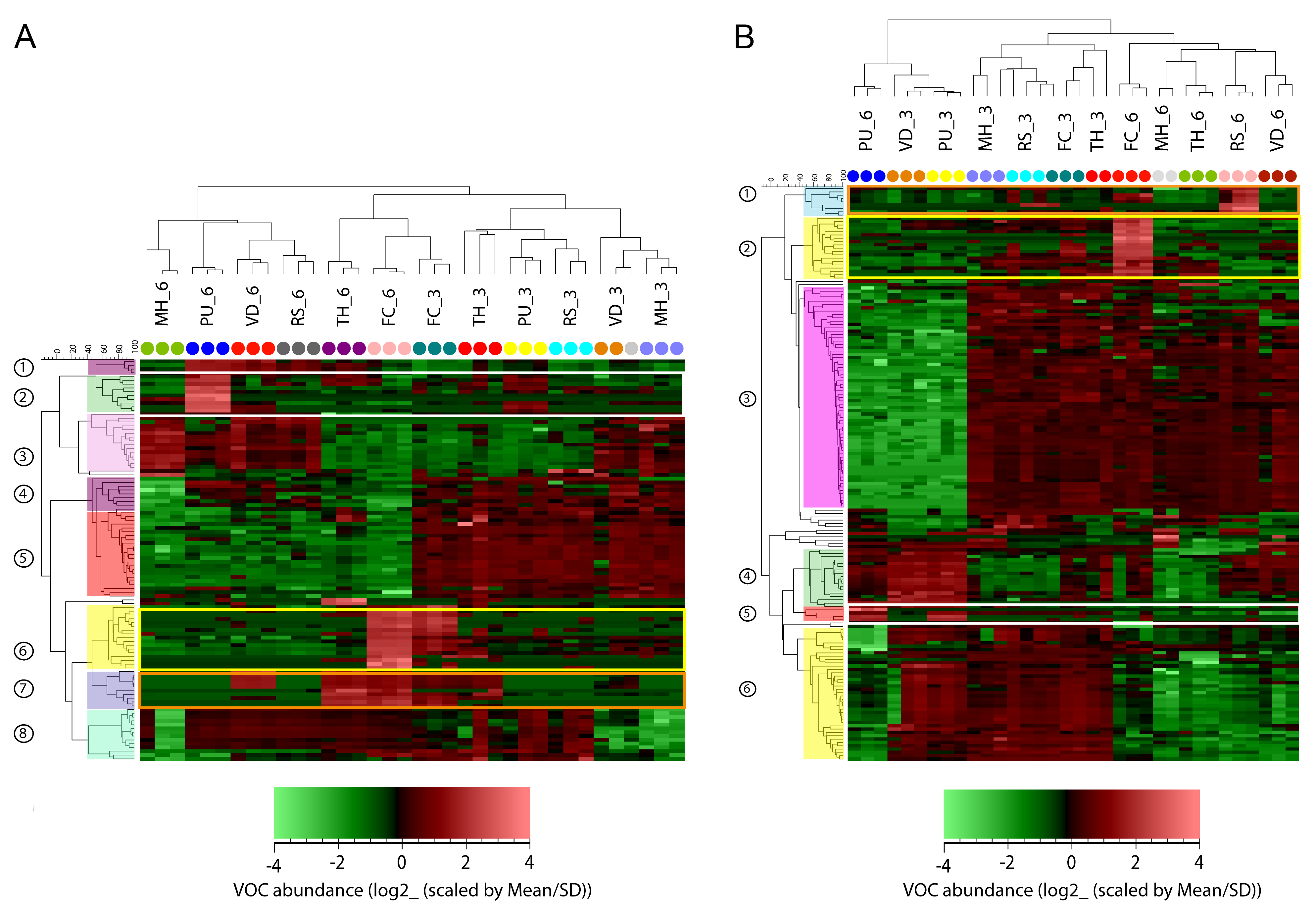

Supplement: FIGURE S2 — Hierarchical cluster analysis (HCA) of fungal and oomycetal strains based on volatiles that were significantly different (P < 0.05 with Bonferroni correction) in abundance between at least two strains when grown on water agar supplied with artificial root exudates (A) and on potato dextrose agar (B) at day 3 (early growth stage) and day 6 (late growth stage). MH, Mucor hiemalis; RS, Rhizoctonia solani; PU, Pythium ultimum; VD, Verticillium dahliae; FC, Fusarium culmorum; TH, Trichoderma harzianum; C, Control (media). The color code below the figure indicates the abundance of the volatiles, which is log2-transformed and scaled by Mean/SD. The boxes indicated in different colors discern the eight distinct clusters that determine the spatial separation of the samples in the PCA. [file Image_2.TIF]

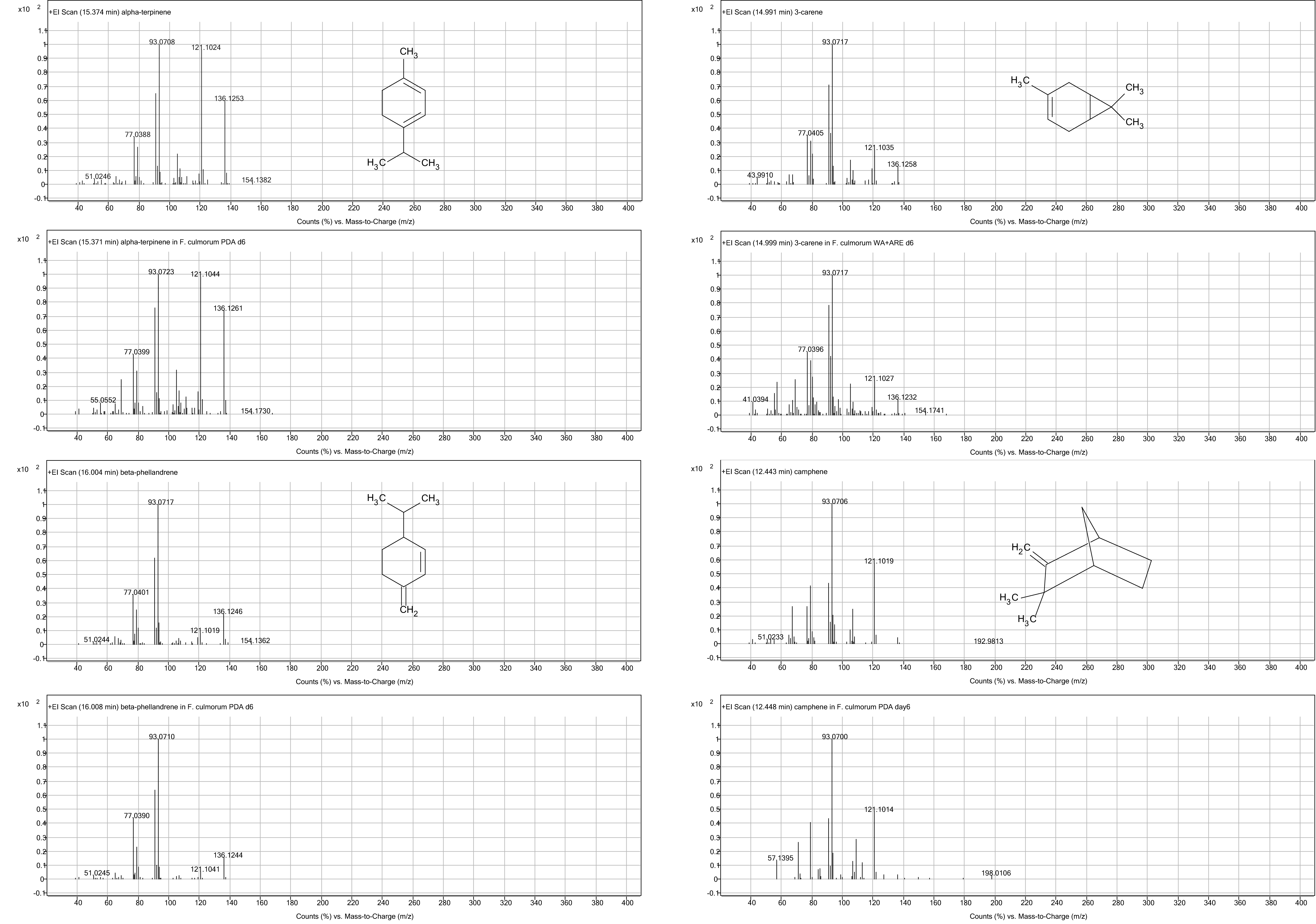

Supplement: FIGURE S3 — Comparison of mass spectra of the pure volatile compounds with those found in F. culmorum. [file Image_3.TIF]
